# Designing TiZrNbTa-Al Medium-Entropy Alloy for Next-Generation Hydrogen Storage

**DOI:** 10.3390/ma19020379

**Published:** 2026-01-17

**Authors:** Jakub Kubaško, Miloš Matvija, Katarína Nigutová, Lenka Oroszová, Zuzana Molčanová, Beáta Ballóková, Róbert Džunda, Gabriel Sučik, Ľuboš Popovič, Róbert Kočiško, Jens Möllmer, Marcus Lange, Karel Saksl

**Affiliations:** 1 Institute of Materials, Faculty of Materials, Metallurgy and Recycling, Technical University of Košice, Letná 9, 042 00 Košice, Slovakia; milos.matvija@tuke.sk (M.M.); gabriel.sucik@tuke.sk (G.S.); lubos.popovic@tuke.sk (Ľ.P.); robert.kocisko@tuke.sk (R.K.); 2 Institute of Materials Research, Slovak Academy of Sciences, Watsonova 47, 040 01 Košice, Slovakia; nigutova@saske.sk (K.N.); loroszova@saske.sk (L.O.); molcanova@saske.sk (Z.M.); bballokova@saske.sk (B.B.); rdzunda@saske.sk (R.D.); 3 Institut für Nichtklassische Chemie e.V. (INC), Permoserstr. 15, D-04318 Leipzig, Germany; moellmer@inc-leipzig.de (J.M.); lange@inc-leipzig.de (M.L.)

**Keywords:** medium-entropy alloy, hydrogen absorption, metal hydride, hydrogen storage

## Abstract

Medium-entropy alloys (MEAs) represent a promising class of materials for solid-state hydrogen storage due to their high hydrogen affinity, structural stability, and tunable properties. In this work, a compositional series of (TiZrNbTa)_{100−x}_Al_x_ (x = 0–10 at. %) MEAs were prepared and systematically investigated to clarify the influence of aluminum addition on microstructure, mechanical response, and hydrogen sorption behavior. The alloys were synthesized by arc melting, homogenized by annealing, and characterized using microscopy, X-ray diffraction, density measurements, microhardness testing, nanoindentation, and hydrogen absorption/desorption experiments. Hydrogen sorption was evaluated by isobaric absorption measurements at 2 MPa H_2_ over two consecutive cycles, complemented by thermogravimetric desorption analysis of hydrogenated samples. The results show that aluminum addition significantly affects activation behavior, hydrogen uptake, and residual hydrogen retention, while simultaneously increasing hardness and elastic modulus in a non-linear manner. The alloy containing 5 at. % Al exhibits the most balanced performance, combining reduced activation temperature in the second absorption cycle, relatively high hydrogen capacity, and moderate mechanical stiffness. These findings demonstrate that controlled aluminum alloying is an effective strategy for tailoring hydrogen–metal interactions and optimizing the performance of TiZrNbTa-based MEAs for solid-state hydrogen storage applications.

## 1. Introduction

The transition toward a sustainable hydrogen-based energy infrastructure requires storage technologies that are efficient, safe, and volumetrically compact. Although hydrogen offers exceptional gravimetric energy density and can be produced renewably, its large-scale deployment is constrained by the limitations of compressed-gas systems, cryogenic liquefaction, and classical intermetallic hydrides, all of which suffer from low volumetric density, energy-intensive operation, and insufficient cycling stability [1,2,3]. These factors have motivated substantial research into solid-state hydrogen-storage materials capable of delivering improved thermodynamic performance, enhanced hydrogen affinity, and reversible sorption behavior under practical conditions [2,4].

In this context, multi-principal element alloy concepts such as medium-entropy alloys (MEAs) and high-entropy alloys (HEAs) represent promising platforms for next-generation hydrogen-storage materials. These systems consist of several principal elements typically present in concentrations between 5 and 35 at. %, enabling configurational entropies of approximately 1.0–1.5 *R* for MEAs and above 1.5 *R* for HEAs [5,6,7], where *R* is the universal (molar) gas constant, *R* = 8.314 J/(mol·K). Their chemical complexity, electronic tunability, and lattice-distortion effects facilitate the formation of simple solid-solution phases (most commonly BCC or FCC), while suppressing brittle intermetallic compounds in many compositions. These features provide unique opportunities to tailor hydrogen–metal interactions, diffusion pathways, and mechanical stability beyond what is achievable in classical binary or ternary hydrides [5,8].

Among these structural possibilities, BCC lattices are particularly advantageous for hydrogen storage, as they offer up to 18 interstitial sites per metal atom (12 tetrahedral and 6 octahedral), compared with only 12 sites in FCC structures (8 tetrahedral and 4 octahedral). Combined with adjustable chemical complexity and local lattice distortions, BCC-structured MEAs and HEAs achieve substantial hydrogen solubility, tunable plateau pressures, and reversible lattice expansion. The literature consistently reports that hydrogen uptake in these materials depends on atomic-size mismatch, mixing enthalpy, valence electron concentration, defect chemistry, and short-range order, with no single descriptor adequately predicting performance across systems [9,10,11,12,13].

Refractory TiZrNbTa MEAs exemplify the advantages of BCC multi-principal alloys for hydrogen applications. Their constituent early transition metals exhibit strong hydride-forming tendencies, and their equiatomic combinations reliably produce stable single-phase BCC solid solutions with high mechanical robustness and thermal stability [14,15,16]. These alloys demonstrate pronounced hydrogen-induced lattice expansion, high solubility, and sorption behavior that is highly sensitive to surface condition, local defect structure, and chemical inhomogeneity [17]. A characteristic feature of TiZrNbTa is its activation-controlled absorption mechanism, where the first hydrogenation cycle requires elevated temperatures due to surface-oxide reduction and initial defect formation, whereas subsequent cycles proceed at markedly lower activation temperatures once vacancy clusters and hydrogen-assisted defect structures facilitate rapid diffusion [17,18]. Subsequent studies have further established that TiZrNbTa-based systems can achieve rapid sorption kinetics and maintain high hydrogen affinity during repeated cycling, underscoring their suitability as base alloys for compositional tuning [19].

Building on this foundation, alloying refractory MEAs with lightweight elements offers a means to tune lattice distortion, hydride stability, and hydrogen–metal interactions. Aluminum has shown particular relevance in this context. In the Ti-V-Nb system, progressive Al substitution decreases dihydride stability, lowers desorption onset temperatures, and modifies plateau behavior while maintaining reversible hydrogen uptake despite a reduction in maximum capacity [20]. Likewise, in quinary Ti-Vr-Nb-M alloys, compositions containing 10 at. % Al remain a single-phase BCC and exhibit among the lowest desorption temperatures within the series, indicating that Al effectively adjusts hydride energetics and diffusion pathways in refractory systems [21]. These observations support the use of Al as a tuning element for refining sorption thermodynamics and kinetics. Motivated by this, the present work examines the (TiZrNbTa)_{100−x}_Al_x_ alloy series to determine how incremental Al additions influence phase stability, microstructure, mechanical properties, and hydrogen absorption behavior. By correlating structural, mechanical, and thermodynamic descriptors across the alloy series, this study aims to clarify the role of Al in modifying hydrogen–metal interactions in refractory MEAs and to identify compositional regimes that optimize activation behavior, gravimetric capacity, and overall suitability for practical solid-state hydrogen-storage applications.

## 2. Materials and Methods

### 2.1. Design of Materials

In order to develop alloys with enhanced hydrogen storage capacity and structural stability, a series of (TiZrNbTa)_{100−x}_Al_x_ (x = 0, 1, 3, 5, 7 and 10 at. %) medium-entropy alloys (MEAs) was designed. The base TiZrNbTa system was selected owing to its high hydrogen affinity, favorable thermodynamic stability, and tendency to form a single-phase BCC (body-centered cubic) solid solution. Aluminum was incorporated as a substituent element to tailor lattice distortion, modify the electronic structure, and influence the hydrogen–metal interaction strength, while preserving solid-solution stability and the refractory BCC matrix [19,22].

The alloy design strategy followed the established Hume–Rothery rules and empirical thermodynamic criteria describing the stability of solid-solution alloys. Solid-solution formation is generally favored when the atomic size difference (*δ*) and mixing enthalpy (Δ*H_mix_*) fall within narrow stability limits (*δ* < 6.6% and −11.6 ≤ Δ*H_mix_* ≤ 3.2 kJ·mol^−1^), while the valence electron concentration (VEC) remains below 6.87, a critical threshold often used to distinguish BCC- from FCC-stabilized solid solutions [23,24].

The characteristic thermodynamic and structural parameters were calculated using the following relations, as summarized in Table 1 [23,24,25]:

(1) $$\delta \;=\;\sqrt{\sum c_{i}{\left(1\;-\frac{r_{i}}{\bar{r}}\right)}^{2}}\times \;100$$

(2) $$VEC=\sum c_{i}{\left(VEC\right)}_{i}$$

(3) $$\Delta H_{mix}=\sum_{i<j}4{\Delta H}_{ij}c_{i}c_{j}$$

(4) $$\Omega =\frac{\left(T_{m}\Delta S_{mix}\right)}{\left|\Delta H_{mix}\right|}$$

where

(5) $$T_{m}=\sum_{i}c_{i}{\left(T_{m}\right)}_{i}$$

and

(6) $$\Delta S_{mix}=-R\;\sum_{i}c_{i}\ln c_{i}$$

In these relations, $r_{i}$ and ${VEC}_{i}$ denote the atomic radius and valence electron concentration of the *i*th element, respectively. The term $\bar{r}=\;\sum c_{i}r_{i}$ represents the composition-weighted average atomic radius. The coefficients $c_{i}$ and $c_{j}$ correspond to the atomic fractions of the constituent elements, while ${\Delta H}_{ij}$ is the enthalpy of mixing between elements *i* and *j* at equimolar concentration in regular binary solutions. The melting temperature of the alloy is obtained as the weighted sum of the melting temperatures of the pure elements ${\left(T_{m}\right)}_{i}$ and the configurational entropy of mixing Δ$S_{mix}$ follows the ideal solution expression while the parameter *Ω* relates the enthalpy of mixing (Δ*H_mix_*), entropy of mixing (Δ*S_mix_*), and the melting temperature (*T_m_*) [23,24,25,26].

Because hydrogen storage behavior strongly depends not only on lattice stability but also on the metal–hydrogen chemical interaction, two additional thermodynamic descriptors, the enthalpy of solution at infinite dilution (Δ*H*_∞_) and the enthalpy of formation of the concentrated hydride (Δ*H_f_*), were also evaluated following the Griessen–Driessen theoretical framework. These parameters qualitatively indicate the alloy’s expected hydrogen affinity and guide the selection of compositions with balanced stability and reversibility [25,26,27].

The hydrogen-affinity descriptors are calculated as follows:

(7) $$\Delta H_{\infty }=\;\sum_{i=i}c_{i}H_{\infty i}$$

where *c_i_* denotes the atomic fraction of the *i*th element, and $H_{\infty i}$ is the enthalpy of solution at infinite dilution for the pure metal *i*, and

(8) $$\Delta H_{f}=\sum_{i=i}c_{i}H_{fi}$$

where $H_{fi}$ represents the enthalpy of formation of the concentrated hydride for the pure metal *i* [25,26,27].

**Figure 1:**
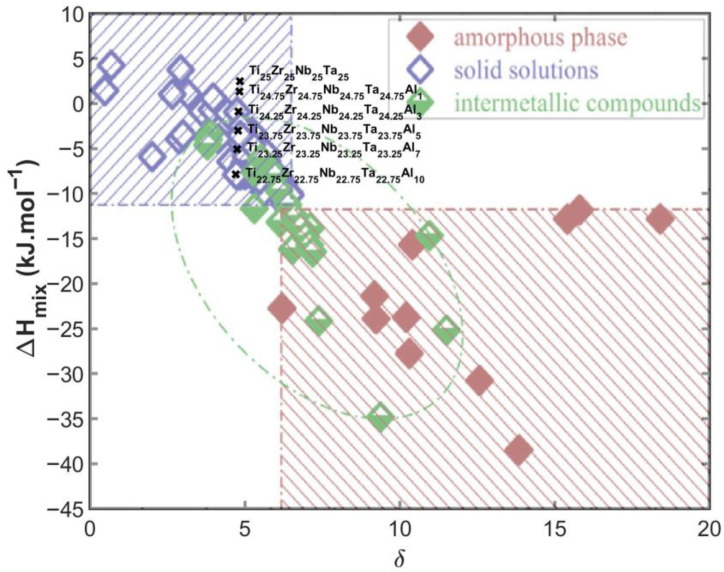
*δ*–Δ*H_mix_* plot delineating the expected phase stability regions for MEAs, highlighting domains associated with solid-solution formation, intermetallic compounds, and amorphous phases. The calculated parameters for the (TiZrNbTa)_{100−x}_Al_x_ alloys plot inside or near the solid-solution region, indicating a strong tendency toward forming a stable BCC solid solution across the entire compositional range [25].

The relationship between *δ* and Δ*H_mix_* is illustrated in Figure 1, where the blue-shaded region corresponds to solid-solution stability, the green ellipse indicates the region of intermetallic compounds, and the red-shaded area denotes amorphous phases. The calculated parameters for the TiZrNbTa–Al system fall within the solid-solution region, confirming the predicted formation of a medium-entropy BCC alloy [25].

From these results, the following conclusions can be drawn:

- All alloys can be classified as medium-entropy alloys (MEAs) since their mixing entropy satisfies 1 *R* < Δ*S_mix_* < 1.5 *R*.
- The Ω parameter > 1.1 for all compositions confirms thermodynamic stability of the solid-solution state.
- Increasing Al content slightly raises *δ* and decreases Δ*H_mix_*, yet all compositions remain within the empirical stability limits (δ ≤ 6.6%, −11.6 ≤ Δ*H_mix_* ≤ +3.2 kJ·mol^−1^).
- The *VEC* values < 4.75 predict a BCC crystal structure, in agreement with the expected phase stability of refractory MEAs [23,24,25].

### 2.2. Material Preparation

The alloys Al-0, Al-1, Al-3, Al-5, Al-7, and Al-10—nominally (TiZrNbTa)_{100−x}_Al_x_ with x = 0, 1, 3, 5, 7, and 10 at. %—were prepared by arc melting under an inert argon atmosphere. High-purity elements were used as initial materials: Ti (99.99%, Alfa Aesar, Ward Hill, MA, USA), Zr (99.2%, Alfa Aesar), Nb (99.9%, chemPUR, Karlsruhe, Germany), Ta (99.95%, Alfa Aesar), and Al (99.9%, Alfa Aesar).

The Ti, Zr, Nb, and Ta elements were weighed according to the target composition and pressed into pellets under a load of approximately 5 tons for 30–60 s to ensure good electrical contact and minimize spattering during melting. The synthesis was performed using the Mini Arc Melting System MAM-1 furnace (Edmund Bühler GmbH, Bodelshausen, Germany) in a high-purity argon atmosphere. A titanium getter was melted before each run to reduce residual oxygen.

The TiZrNbTa base alloy was melted and remelted six times, with the button inverted after each melting step to promote chemical homogeneity. After homogenizing the base alloy, the required Al pieces were added directly onto the button without any additional pressing and the alloys were remelted four more times under argon, turning the loaf upside down between each melt. The total mass loss during melting was below 1%.

The as-cast alloys were subsequently subjected to a homogenization heat treatment at 1200 °C for 24 h in a tubular furnace under flowing argon, followed by water quenching to retain the high-temperature microstructure. The heat-treated samples were then prepared for microstructural, mechanical, and hydrogen-sorption characterization.

### 2.3. Characterization of Materials

The density of the alloys was determined by the Archimedes method using the precision balance Kern ABT 120-4M (KERN & SOHN GmbH, Balingen, Germany) equipped with the adapter ABT-A01 for density measurement. Reported values represent the average of ten independent measurements, with a deviation below 0.5%.

For microstructural and chemical characterization, an alloy sample was mounted in an electrically conductive resin Polyfast (Struers, Ballerup, Denmark) for metallographic analysis. Mounted samples were ground, polished, and etched following standard metallographic procedures. Optical microscopy was performed on etched surfaces using the Olympus Vanox-T AH-2 light microscope (Olympus Corporation, Tokyo, Japan) to examine the general morphology and grain features. For phase identification and hydrogen-sorption experiments, a sample was mechanically pulverized into a fine powder. Powder preparation was carried out by mechanical milling for approximately 30 min under Ar atmosphere, followed by sieving to obtain the <40 µm fraction.

Detailed microstructural observations were carried out on etched surfaces using the JEOL JSM-7000F scanning electron microscope (JEOL Ltd., Tokyo, Japan) equipped with secondary-electron (SE) detector and backscattered-electron (BSE) detector for phase-contrast imaging. Energy-dispersive X-ray spectroscopy (EDS) was performed on the polished metallographic cross-sections of all (TiZrNbTa)_{100−x}_Al_x_ alloys to evaluate the chemical composition. For each specimen, five area scans were collected at a magnification of 200× from different specimen regions. The average composition and corresponding standard deviation were calculated for each alloy to obtain representative chemical data.

The microhardness was measured on polished samples using the Wilson–Wolpert Tukon 1102 hardness tester (Berg Engineering & Sales Co., Inc., Rolling Meadows, IL, USA) equipped with a Vickers indenter. Measurements were performed under a load of 0.2 kgf (HV0.2) with a dwell time of 10 s. Fifteen indentations were made for each composition and the mean value with standard deviation was calculated.

The nanoindentation measurements were carried out using the TTX-NHT instrument (CSM Instruments, Peseux, Switzerland) based on the depth-sensing indentation (DSI) technique. A Berkovich-pyramid diamond tip was used in linear-mode loading at a frequency of 10 Hz. The applied load was 80 mN, with loading and unloading rates of 200 mN min^−1^ and a 10 s hold time at maximum load. The resulting load–penetration (P–h) curves were analyzed according to the Oliver and Pharr method [28] to determine hardness and elastic modulus as functions of indentation depth. Up to 20 indentations were performed for each alloy and the results were statistically evaluated.

For phase analysis, X-ray diffraction (XRD) measurements were performed on the powdered specimens using the Philips X’Pert PRO diffractometer (Malvern Panalytical, Almelo, The Netherlands) with Co Kα radiation (λ = 1.78897 Å). Data were collected in the 2θ range of 20–120°, with a step size of 0.03° and a counting time of 30 s per step.

### 2.4. Hydrogen Sorption Experiments

Hydrogen absorption and desorption measurements were carried out using the magnetic suspension balance (IsoSORP series, TA Instruments, New Castle, DE, USA), which operates at pressures up to 50 MPa with a measurement accuracy of 0.05%. The hydrogen content was determined gravimetrically from the buoyancy-corrected mass changes of the sample according to the method described by Moellmer et al. [29]. For this purpose, the skeletal density was calculated from the experimentally measured bulk density, assuming a non-porous sample.

The experimental procedure followed these steps:

1. Approximately 0.5 g of alloy powder was placed into the reaction chamber of the magnetic suspension balance. The system was then evacuated using a rotary vacuum pump to a pressure below 1 Pa to remove residual gases and moisture.
2. The chamber was filled with hydrogen to a pressure of 2 MPa and the initial mass of the sample was recorded at 50 °C under this hydrogen pressure.
3. The chamber temperature (and thus the sample temperature) was increased from room temperature to 300 °C in 25 °C increments. At each step, the sample mass was monitored for 10 min, with each temperature segment lasting approximately 20–30 min in total. This isobaric measurement identified the temperature at which the alloy began to significantly absorb hydrogen.
4. After completing the isobaric cycle, the sample was cooled to 50 °C while maintaining the hydrogen pressure of 2 MPa. A subsequent mass measurement was performed at this temperature to determine the total amount of absorbed hydrogen.
5. The chamber was then evacuated and heated to 400 °C for 3 h to desorb hydrogen from the sample.
6. Steps 2–4 were repeated once more to verify reproducibility and assess the cyclic stability of the absorption–desorption behavior.

After hydrogen cycling, the powders were recovered and stored under an argon atmosphere for subsequent X-ray diffraction (XRD). The hydrogen-to-metal ratio (H/M) was calculated from the measured mass change, considering buoyancy corrections and the alloy’s molar mass [29].

The amount of remaining chemically bound hydrogen in the alloys after the sorption experiment was determined by simultaneous thermal analysis (STA) using the thermogravimetric (TG) method. The measurements were performed using an STA 449 F1 Jupiter system (NETZSCH, Gerätebau, Germany) at a heating rate of 10 K·min^−1^ up to 900 °C. The analysis was conducted on powder samples with a mass of approximately 100 mg under a high-purity nitrogen atmosphere.

## 3. Results and Discussion

The outcomes of the material characterization for the (TiZrNbTa)_{100−x}_Al_x_ alloys with x = 0, 1, 3, 5, 7, and 10 at. % are summarized in Table 2.

### 3.1. Chemical Composition and Density

EDX spectroscopy performed on polished metallographic cross-sections of the (TiZrNbTa)_{100−x}_Al_x_ alloys confirmed a chemical composition close to the nominal values. For all compositions, the maximum deviation did not exceed ±2 at. %, which is consistent with typical arc-melted materials and comparable to deviations reported in similar HEA/MEA systems [17,19,20,21]. The measured densities exhibited only minor variation across the alloy series, reflecting the comparable atomic packing and similar elemental ratios. As aluminum was incrementally introduced at the expense of heavier refractory elements (Ti, Zr, Nb, and Ta), a slight but systematic decrease in density was observed—from 8.85 g·cm^−3^ for Al-0 to 8.26 g·cm^−3^ for Al-10. This trend is fully expected, given the relatively small substitutional shift in overall composition.

### 3.2. Microstructure

**Figure 2:**
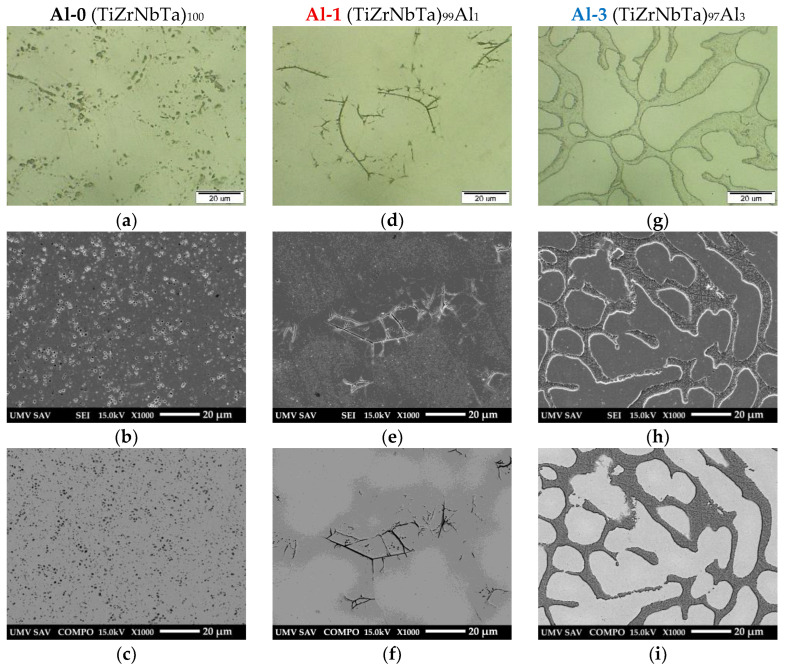
Microstructure of Al-0 (**a**–**c**), Al-1 (**d**–**f**), and Al-3 (**g**–**i**) alloys documented by light microscope and scanning electron microscope using SE and BSE detection.

**Figure 3:**
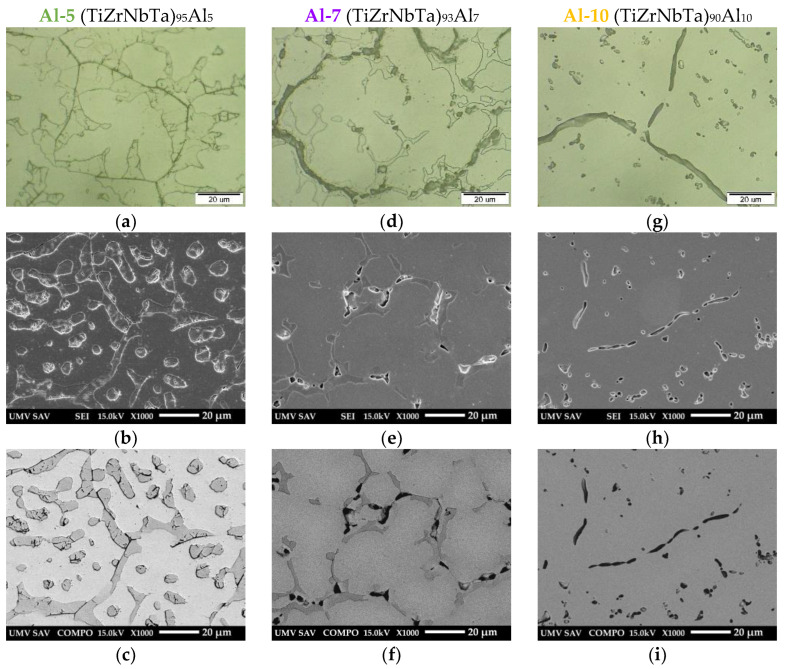
Microstructure of Al-5 (**a**–**c**), Al-7 (**d**–**f**) and Al-10 (**g**–**i**) alloys documented by light microscopy and scanning electron microscopy using SE and BSE detection.

The microstructures of the alloys Al-0, Al-1, and Al-3 are shown in Figure 2, while those of Al-5, Al-7, and Al-10 are presented in Figure 3. The microstructure of the analyzed alloys was complex and heterogeneous, consisting of dendrites, granular features, and intermetallic particles of various morphologies predominantly located in the interdendritic regions.

A comparison of the microstructures in Figure 2 and Figure 3 clearly reveals the influence of aluminum content on the microstructural characteristics of the alloys. It is evident that increasing Al content resulted in enhanced microstructural heterogeneity and promoted the formation of intermetallic phase particles.

EDS analyses confirmed that the solid solution contained the major chemical elements and the corresponding Al content for each analyzed alloy, with the exception of the Al-0 alloy. The intermetallic particles were of different types. In the Al-0 alloy, needle-like and rod-like particles were observed, enriched in Ti and Zr and containing lower contents of Nb and Ta. The oval particles consisted mainly of Nb, Zr, and Ti, with a minor content of Zr. In the Al-3 alloy, a pronounced irregular phase was present in the interdendritic regions, predominantly enriched in Zr, Ti, Nb, and Ta, with a minor Al content. The microstructure of the Al-5 alloy contained both oval and irregular particles, rich in Zr, Nb, Ti, and Ta, and also containing minor contents of Al. The oval particles were mainly composed of Nb, Ta, and Ti, with Zr present only in minor quantities. In the case of the Al-7 alloy, two types of intermetallic particles with irregular morphology were identified as follows: particles enriched in Zr, Ti, Nb, and Ta with a corresponding content of Al, and particles primarily enriched in Ta, Nb, and Ti with a corresponding Al content but with a reduced Zr content. The microstructure of the Al-10 alloy contained oval and globular particles enriched in Ti, Nb, Ta, Zr, and corresponding levels of Al, as well as elongated particles located at the boundaries of structural features, enriched in Zr and Al and containing lower amounts of Ti, Nb, and Ta.

### 3.3. Mechanical Properties

**Figure 4:**
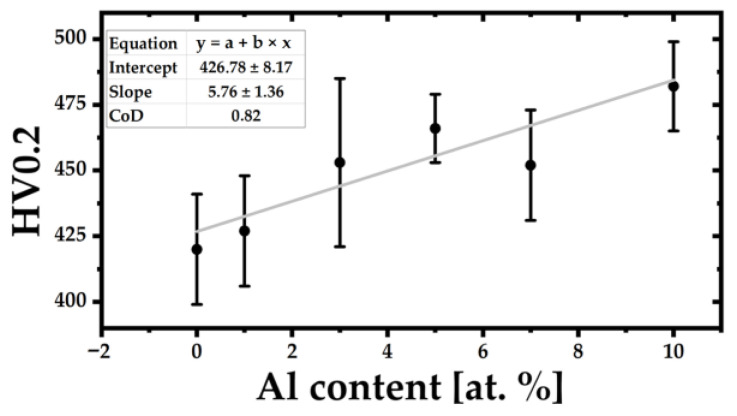
Dependence of microhardness HV0.2 on Al content in the (TiZrNbTa)_{100−x}_Al_x_ alloys.

**Figure 5:**
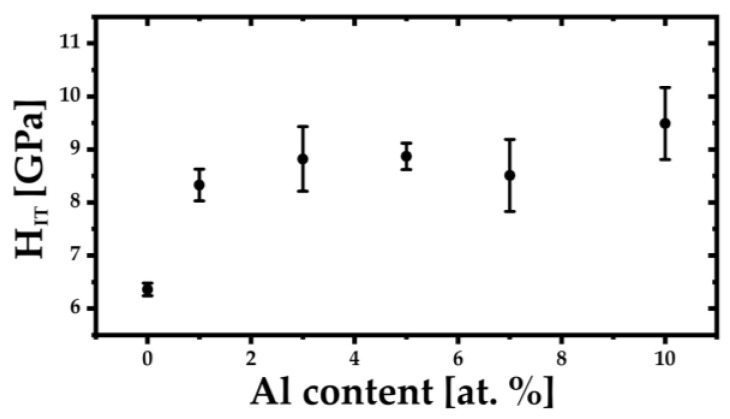
Dependence of nanohardness H_IT_ on Al content in the (TiZrNbTa)_{100−x}_Al_x_ alloys.

**Figure 6:**
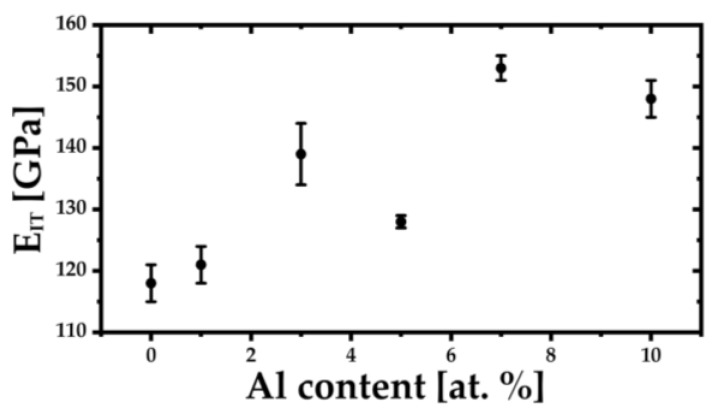
Dependence of elastic modulus E_IT_ on Al content in the (TiZrNbTa)_{100−x}_Al_x_ alloys.

The mechanical properties of the (TiZrNbTa)_{100−x}_Al_x_ alloys were evaluated using the Vickers microhardness (HV0.2) and instrumented nanoindentation. The results are summarized in Table 2, while the corresponding compositional trends are illustrated in Figure 4, Figure 5 and Figure 6. This shows that the addition of aluminum to the TiZrNbTa alloy played a positive role in terms of hardness and elasticity.

The microhardness increases progressively from Al-0 to Al-5, rising from 420 HV to 466 HV, as shown in Figure 4. This linear increase reflects the strengthening effect of Al on the BCC solid solution, caused by enhanced lattice distortion and increased resistance to plastic deformation. A small decrease for Al-7, followed by a renewed increase for Al-10, indicates that the strengthening mechanism becomes non-linear at higher Al concentrations. The decrease in the HV0.2 hardness value observed for the Al-7 alloy was caused by a significant change in the alloy’s microstructural character compared with the Al-5 alloy. A comparison of their microstructures shows that the Al-7 alloy exhibited larger regions with coarser solid-solution grains than the Al-5 alloy, which likely resulted in the slightly lower HV0.2 hardness measured.

A similar compositional dependence is observed for the nanohardness H_IT_, presented in Figure 5. The indentation hardness increases from 6.4 ± 0.1 GPa for Al-0 to 8.9 ± 0.3 GPa for Al-5, indicating a pronounced strengthening effect at the nanoscale with increasing Al content. With further Al addition, H_IT_ remains at elevated levels, reaching 8.5 ± 0.7 GPa for Al-7 and the highest value of 9.5 ± 0.7 GPa for Al-10. The overall correspondence between HV0.2 and H_IT_ trends confirms that the observed strengthening reflects intrinsic bulk behavior rather than surface-related artifacts.

The indentation modulus E_IT_, shown in Figure 6, displays a more complex compositional dependence. The modulus increases from 118 GPa for Al0-Z to 139 GPa for Al3-Z, followed by a distinct drop at Al5-Z (128 GPa). To exclude surface artifacts or localized microstructural effects, the Al5-Z specimen was repolished and remeasured in a different region. An additional set of 20 indentation imprints reproduced the modulus decrease, confirming that this behavior is an intrinsic material property. At higher Al contents, the modulus rises sharply to 153 GPa for Al7-Z and slightly decreases to 148 GPa for Al10-Z.

The correlations between mechanical and hydrogen sorption properties (Section 3.4) are evident. Alloys exhibiting higher hardness and elastic modulus values, particularly Al-7 and Al-10, show reduced hydrogen uptake and higher activation temperatures, consistent with the concept that increased lattice stiffness and resistance to deformation hinder hydrogen adsorption and diffusion. Conversely, Al-5 combines moderate hardness and elastic modulus with favorable hydrogen absorption behavior, suggesting an optimal balance between mechanical stability and lattice flexibility. This trend is consistent with observations by Varcholová et al., who reported that increasing hardness in BCC MEA systems leads to a reduction in hydrogen storage capacity due to suppressed hydrogen mobility within the lattice [30].

### 3.4. Hydrogen Sorption Properties of Materials

**Figure 7:**
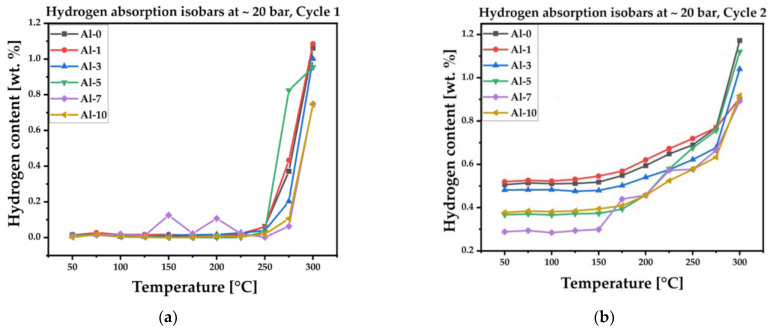
Hydrogen absorption behavior of the (TiZrNbTa)_{100−x}_Al_x_ alloys under constant pressure and increased temperature for (**a**) the 1st absorption cycle and (**b**) the 2nd absorption cycle (isobars).

Hydrogen sorption behavior of the (TiZrNbTa)_{100−x}_Al_x_ alloys was investigated using isobaric absorption measurements at 2 MPa H_2_, which corresponds to the operating conditions of low-pressure metal hydride storage systems. The alloys were heated in incremental steps from 50 °C to 300 °C, and the gravimetrically determined mass change was used to evaluate both the onset of hydrogen absorption and the maximum storage capacity. Two consecutive absorption cycles were performed to assess activation behavior, stability, and the reproducibility of the hydrogen uptake process. As shown in Figure 7a,b, the first absorption cycle provides information about the initial activation temperature and the intrinsic hydrogen affinity of each alloy, while the second cycle reflects the behavior after surface activation and the establishment of hydrogen diffusion pathways. The results of both cycles, together with the corresponding hydrogen-to-metal ratios (H/M), activation temperatures, and related material parameters, are summarized in the Table 2.

The hydrogen sorption measurements were performed under isobaric conditions at 2 MPa H_2_ to enable consistent comparison of activation behavior and hydrogen uptake across all investigated compositions. Although this approach does not provide full thermodynamic information such as plateau pressures from PCT analysis, it is well suited for comparative screening of multi-component alloy systems. Detailed PCT measurement of selected compositions are planned as future work to fully assess hydride thermodynamics.

Analysis of the isobaric absorption curves indicates several distinct behaviors among the studied alloys:

- The base alloy Al-0 begins to absorb hydrogen only above ~275 °C in the first cycle. Its maximum storage capacity reaches 1.06 wt. % (H/M = 1.09) in Cycle 1 and increases to 1.17 wt. % (H/M = 1.21) in Cycle 2, indicating improved activation after the initial hydrogenation.
- The alloy Al-1 activates at a slightly lower temperature of ~250 °C during the first cycle. The maximum absorbed hydrogen amount is 1.08 wt. % (H/M = 1.14) in Cycle 1. In Cycle 2, absorption already begins above ~175 °C, although the maximum capacity decreases to 0.91 wt. % (H/M = 0.95).
- The alloy Al-3 shows activation at temperatures above ~275 °C in Cycle 1. The maximum hydrogen content is 1.00 wt. % (H/M = 1.01), increasing to 1.04 wt. % (H/M = 1.05) in Cycle 2, where the activation temperature shifts to ~200 °C.
- With 5 at. % Al (Al-5), hydrogen absorption starts above ~250 °C in Cycle 1, reaching 0.95 wt. % (H/M = 0.95). In Cycle 2, activation shifts further down to ~200 °C, and the maximum capacity increases to 1.12 wt. % (H/M = 1.12).
- The alloy Al-7 requires temperatures above ~275 °C to activate in the first cycle, and the maximum absorbed amount remains lower, at 0.75 wt. % (H/M = 0.73). During the second cycle, absorption begins above ~250 °C, with a maximum capacity of 0.89 wt. % (H/M = 0.88).
- Further increasing the aluminum content to 10 at. % (Al-10) maintains a high activation temperature of ~275 °C in Cycle 1. The maximum hydrogen content is 0.74 wt. % (H/M = 0.71), improving to 0.92 wt. % (H/M = 0.89) in Cycle 2, where absorption again begins above ~250 °C.

After the first absorption–desorption cycle, all alloys retained a small fraction of chemically bonded hydrogen that did not fully desorb. This is apparent from the second-cycle curves, where the initial hydrogen content is above zero for all compositions. The lowest residual amounts appear in Al-7 and Al-5, whereas Al-0 and Al-1 exhibit the highest retained hydrogen at the beginning of Cycle 2. Based on the overall activation behavior, hydrogen uptake, and the shift in activation temperature between cycles, the alloy Al-5 appears to offer the most balanced performance among the studied compositions. However, additional cycling experiments are required to determine whether the observed decrease in activation temperature continues with further cycles and to evaluate the long-term stability of hydrogen absorption and desorption behavior. The hydrogen storage capacities obtained in this study fall within the range typically reported for refractory BCC HEA/MEA hydrides, while Al addition primarily acts as a tuning element that introduces a trade-off between maximum gravimetric capacity and improved activation behavior and reversibility [16,17,18,19,20,21]. To further verify the presence of residual hydrogen, desorption measurements were also performed after the second cycle.

**Figure 8:**
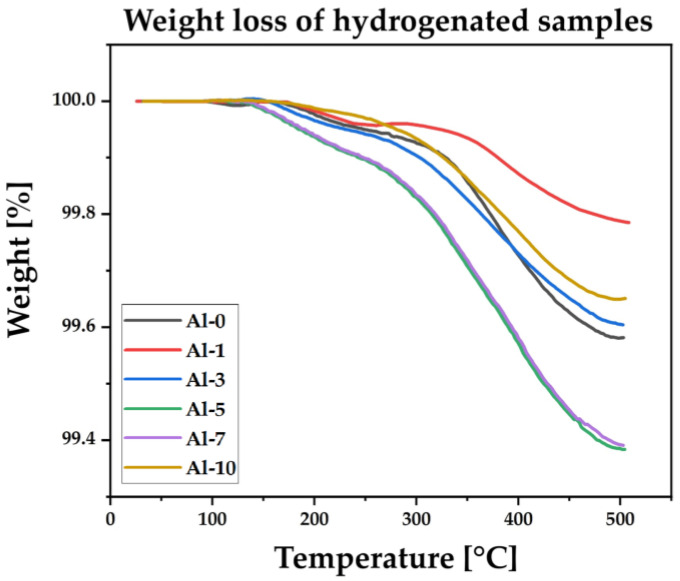
Thermogravimetric desorption measurements of the (TiZrNbTa)_{100−x}_Al_x_ alloys at a heating rate of 10 K·min^−1^.

Hydrogen desorption behavior of the (TiZrNbTa)_{100−x}_Al_x_ alloys was evaluated to determine the stability of the hydrogenated alloys and the extent of residual hydrogen trapping. The measured residual hydrogen contents are listed in Table 2, and the corresponding thermogravimetric (TG) desorption curves are shown in Figure 8. After completion of the absorption sequence, all samples were analyzed in their fully hydrogenated state, and mass loss associated with hydrogen release was recorded at heating rate of 10 K·min^−1^ in nitrogen atmosphere.

Hydrogen desorption in all samples begins above 150 °C, as revealed by the TG curves in Figure 8. An initial decrease in mass is observed at distinct temperatures across the alloy series: for the Al-5 and Al-7 alloys, the onset of measurable desorption occurs at approximately 150–160 °C, whereas for the remaining compositions the desorption onset appears later, within the 180–200 °C range. Beyond these temperatures, the mass loss increases progressively throughout the TG heating process. During the measurement, the Al-0 alloy releases 0.42 wt. % hydrogen, while the Al-1 alloy releases 0.22 wt. %, the lowest value observed in the series. The Al-3 alloy shows a total hydrogen release of 0.40 wt. %, followed by the Al-5 alloy, which exhibits the highest release of 0.62 wt. %. A comparable amount, 0.61 wt. %, is released from the Al-7 alloy. For the Al-10 composition, the total hydrogen release reaches 0.35 wt. %. Across all alloys, the principal mass-loss region occurs at temperatures well above the initial onset (150–200 °C), indicating that the majority of the released hydrogen is removed only at higher temperatures during the TG run.

### 3.5. Phase Analysis

**Figure 9:**
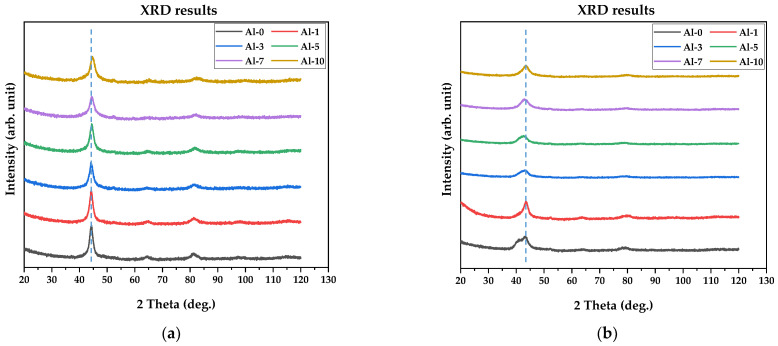
X-ray diffraction (XRD) patterns of the annealed (TiZrNbTa)_{100−x}_Al_x_ alloys measured in powder form (**a**) before absorption, and (**b**) after the 2nd hydrogen absorption cycle. The patterns are vertically offset for clarity.

Phase analysis was performed on all (TiZrNbTa)_{100−x}_Al_x_ alloys in powder form using X-ray diffraction. Measurements were carried out for samples in the annealed state before hydrogen absorption and after the second hydrogen absorption cycle. The XRD patterns of the annealed samples are shown in Figure 9a,b.

Despite long-term homogenization annealing (1200 °C, 24 h), the XRD patterns of the as-prepared alloys exhibit pronounced Bragg-peak broadening. In agreement with the microstructural observations, the broad maxima are superpositions of reflections from multiple BCC phases (space group Im-3m, No. 229) with slightly different lattice parameters arising from subtle chemical partitioning. Therefore, the prepared alloys should be regarded as multiphase rather than strictly single-phase solid solutions. From the position of the dominant fundamental BCC reflection (see note below), the average lattice parameter can be estimated as a ≈3.36 Å, which is very close to that reported for the intermetallic phase (NbTiZr)_0.66_ (COD entry No. 96-153-7634).

After two consecutive hydrogen absorption cycles, all alloys become even less crystalline, as indicated by a further increase in peak broadening and markedly reduced intensities of the remaining Bragg reflections. The alloys with lower Al content (Al-1 and Al-3) show a discernible splitting of the first BCC peak, consistent with the formation of an additional hydride-related contribution. In contrast, the Al-5 and Al-7 alloys show the most pronounced shift of the dominant BCC reflections toward lower 2θ angles, corresponding to an expanded lattice parameter of approximately a ≈3.50 Å. This provides clear evidence for the largest lattice expansion upon hydrogen uptake and, consequently, the highest amount of retained (chemically bound) hydrogen. The dominant peak position of Al-10 shifts less than that of Al-5 and Al-7, indicating lower hydrogen retention after two absorption cycles. This trend is fully consistent with the residual-hydrogen contents determined by the STA/TG desorption experiments.

## 4. Conclusions

In this work, a compositional series of (TiZrNbTa)_{100−x}_Al_x_ medium-entropy alloys was prepared and systematically studied in terms of microstructural properties, mechanical response, and hydrogen sorption behavior. Incremental substitution of aluminum in the refractory TiZrNbTa base alloy leads to pronounced changes in hydrogen absorption characteristics, including activation temperature, maximum storage capacity, and residual hydrogen content after repeated cycling.

X-ray diffraction (XRD) indicates that, even after homogenization (1200 °C/24 h), the alloys are not strictly single-phase. The as-prepared state is characterized by broadened Bragg maxima consistent with a predominantly BCC (Im-3m) multiphase constitution with slight lattice-parameter variations arising from chemical heterogeneity. Upon hydrogen cycling, all compositions exhibit further peak broadening and intensity reduction, evidencing decreased crystallinity. The alloys with intermediate Al contents (notably Al-5 and Al-7) show the most pronounced shift of reflections toward lower 2θ angles, demonstrating the largest lattice expansion and thus the highest degree of hydrogen-induced structural dilation, whereas Al-10 exhibits a smaller shift, consistent with reduced hydrogen retention.

Mechanical testing reveals an overall increase in microhardness and nanohardness with increasing Al content, reflecting the strengthening effect of aluminum in the BCC solid solution. This trend indicates enhanced resistance to plastic deformation induced by Al addition. In contrast, the elastic modulus exhibits a comparatively weaker compositional dependence, showing an overall increasing tendency accompanied by noticeable scatter. This behavior suggests that aluminum addition predominantly affects plastic deformation mechanisms, while elastic lattice stiffness is less sensitive to Al content and is additionally influenced by local compositional variations and microstructural heterogeneity inherent to multicomponent alloys.

Hydrogen absorption measurements show that aluminum additions modify both the activation behavior and the achievable hydrogen capacity, with the most favorable combination observed for the alloy containing 5 at. % Al. This composition exhibits a reduced activation temperature in the second absorption cycle (≈200 °C), a relatively high hydrogen uptake of 1.12 wt. % (H/M ≈ 1.12), and moderate mechanical stiffness (HV0.2 ≈ 466, E_IT_ ≈ 128 GPa) compared with alloys containing higher Al concentrations. Thermogravimetric desorption experiments further indicate that the amount of chemically bound hydrogen depends strongly on alloy composition, reaching a maximum at intermediate Al contents and decreasing at both lower and higher Al levels. Detailed PCT measurements of selected compositions are required in future work to fully establish hydride thermodynamics and long-term storage performance.

In addition, future studies will focus on a more detailed assessment of the role of microstructural state and defect-related effects on hydrogen sorption behavior. In particular, a systematic comparison between as-cast and heat-treated conditions will be performed to clarify the influence of thermal processing on phase stability, hydrogen uptake, and reversibility, and to evaluate whether homogenization heat treatment is essential for achieving optimal hydrogen storage performance in this alloy system.

Overall, the results demonstrate that aluminum is an effective alloying element for tuning the hydrogen sorption behavior of TiZrNbTa-based medium-entropy alloys. The observed correlations between mechanical response, hydrogen uptake and residual hydrogen retention highlight the importance of compositional optimization for the design of refractory MEAs suitable for solid-state hydrogen storage applications.

## Figures and Tables

**Table 1:** Thermodynamic parameters of the alloy (TiZrNbTa)_{100−x}_Al_x_ investigated.

Alloy	δ [%]	Δ*H_mix_* [kJ·mol^−1^]	Δ*S_mix_* [J·K^−1^·mol^−1^]	Ω	*T_m_* [K]	*VEC*	Δ*H*_∞_	Δ*H_f_*
**Al-0** (TiZrNbTa)_100_	4.84	+2.50	1.39 R	11.60	2517.0	4.50	−44.50	−58.00
**Al-1** (TiZrNbTa)_99_Al_1_	4.83	+1.35	1.43 R	21.98	2501.2	4.49	−43.46	−57.39
**Al-3** (TiZrNbTa)_97_Al_3_	4.80	−0.88	1.48 R	34.60	2469.5	4.46	−41.28	−56.17
**Al-5** (TiZrNbTa)_95_Al_5_	4.78	−3.02	1.52 R	10.18	2437.8	4.43	−39.13	−54.95
**Al-7** (TiZrNbTa)_93_Al_7_	4.75	−5.06	1.54 R	6.10	2406.1	4.39	−36.98	−53.73
**Al-10** (TiZrNbTa)_90_Al_10_	4.71	−7.87	1.57 R	3.92	2360.2	4.35	−33.86	−51.96

**Table 2:** Results from the measurement of Al-modified (TiZrNbTa)_{100−x}_Al_x_ alloys. Chemical composition of the alloys determined by the EDX spectroscopy, density, microhardness HV0.2, nanoindentation hardness H_IT_, elastic modulus, activation temperature of hydrogen absorption at pressure of 20 bar for the 1st and 2nd cycle, maximum achieved storage capacity of hydrogen in the alloy for the 1st and 2nd cycle, and the amount of residual hydrogen chemically bounded to the alloy matrix after the 2nd cycle.

Alloy *EDX Composition* [at. %]	Density ρ [g·cm^−3^]	Hardness HV0.2	Nanoindentation H_IT_ [GPa]	Elastic Modulus E [GPa]	Act. Temp. 1st Cycle T_A_ [°C]	Act. Temp. 2nd Cycle T_A_ [°C]	Max. H_2_ Cap. 1st Cycle [wt. %] (*H*/*M*)	Max. H_2_ Cap. 2nd Cycle [wt. %] (*H*/*M*)	Residual H_2_ Content [wt. %]
**Al-0** (TiZrNbTa)_100_ *Ti_23_Zr_26_Nb_27_Ta_24_*	8.85 ± 0.02	420 ± 21	6.4 ± 0.1	118 ± 3	>275	>200	**1.06** *(1.09)*	**1.17** *(1.21)*	0.42
**Al-1 **(TiZrNbTa)_99_Al_1_ *Ti_23_Zr_25_Nb_25_Ta_26_Al_1_*	8.74 ± 0.04	427 ± 21	8.3 ± 0.3	121 ± 3	>250	>175	**1.08** *(1.14)*	**0.91** *(0.95)*	0.22
**Al-3** (TiZrNbTa)_97_Al_3_ *Ti_22_Zr_26_Nb_26_Ta_23_Al_3_*	8.60 ± 0.05	453 ± 32	8.8 ± 0.6	139 ± 5	>275	>200	**1.00** *(1.01)*	**1.0**4 *(1.05)*	0.40
**Al-5** (TiZrNbTa)_95_Al_5_ *Ti_22_Zr_25_Nb_25_Ta_23_Al_5_*	8.57 ± 0.05	466 ± 13	8.9 ± 0.3	128 ± 1	>250	>200	**0.95** *(0.95)*	**1.12** *(1.12)*	0.62
**Al-7** (TiZrNbTa)_93_Al_7_ *Ti_22_Zr_24_Nb_24_Ta_23_Al_7_*	8.28 ± 0.02	452 ± 21	8.5 ± 0.7	153 ± 2	>275	>250	**0.75** *(0.73)*	**0.89** *(0.88)*	0.61
**Al-10** (TiZrNbTa)_90_Al_10_ *Ti_21_Zr_25_Nb_23_Ta_22_Al_9_*	8.26 ± 0.02	482 ± 17	9.5 ± 0.7	148 ± 3	>275	>250	**0.74** *(0.71)*	**0.92** *(0.89)*	0.35

## Data Availability

The original contributions presented in this study are included in the article. Further inquiries can be directed to the corresponding authors.

## Abbreviations

The following abbreviations are used in this manuscript:

MEA	Medium-Entropy Alloy
HEA	High-Entropy Alloy
BCC	Body-Centered Cubic
FCC	Face-Centered Cubic
VEC	Valence Electron Concentration
SE	Secondary Electrons
BSE	Backscattered Electrons
EDS	Energy-Dispersive X-ray Spectroscopy
HV0.2	Vickers hardness (test load 0.2 kgf)
XRD	X-ray Diffraction
H/M	Hydrogen-to-Metal atomic ratio
STA	Simultaneous Thermal Analysis
TG	Thermogravimetric Analysis
H_IT_	Indentation hardness (nanoindentation)
E_IT_	Elastic modulus from nanoindentation
PCT	Pressure-Composition-Temperature
COD	Crystallography Open Database
